# Modelling impairment of evoked gamma range oscillations in schizophrenia

**DOI:** 10.1186/1471-2202-16-S1-P305

**Published:** 2015-12-18

**Authors:** Christoph Metzner, Achim Schweikard, Bartosz Zurowski

**Affiliations:** 1 Institute for Robotics and Cognitive Systems, University of Luebeck, 23538 Luebeck, Germany; 2 Department of Psychiatry, University of Luebeck, Schleswig-Holstein, 23538 Luebeck, Germany

## 

Abnormal oscillatory activity in schizophrenia has been found in a wide range of experimental paradigms [1]. For example, schizophrenic patients show reduced evoked gamma activity, which has been associated with negative symptoms, and increased spontaneous gamma activity, which has been associated with positive symptoms [2]. However, the underlying mechanisms remain elusive. Here we investigated the impact of circuit abnormalities on oscillatory activity in the gamma range (> 30 Hz) by simulating auditory entrainment in an established computational model of the primary auditory cortex [3]. Auditory click entrainment experiments showed that for schizophrenic patients EEG/MEG power decreased at 40 Hz and increased at 20 Hz in response to 40 Hz drive but no differences between were visible in response to 30 Hz drive [4,5].

Here we used the primary auditory cortex model from Beeman [3] and simulated click train stimulation at 40 Hz, to investigate gamma entrainment deficits, and at 30 Hz as a control condition. Without alterations the model entrained at the driving frequency of 30 and 40 Hz, respectively. Similar to previous approaches [6], however, focusing on evoked rather than spontaneous activity, we next explored the effects of (1) connectivity disturbances (reduced (a) recurrent excitation, (b) pyramidal cell input and (c) total connectivity), (2) prolonged GABAergic decay time constant, and (3) reduced inhibitory output.

All three interventions in connectivity (1a-c) led to an increase in 40 Hz power for 40 Hz drive, contrary to human EEG/MEG experiments. A prolonged GABAergic decay time constant produced a reduction of power at 40 Hz and an increase in power at 20 Hz, for the 40 Hz drive, which concurs with [4,5]. Furthermore, for the 30 Hz drive, no differences to the standard model were observed. Reduction of inhibitory output led to decreases in power at 40 Hz for 40 Hz drive but no increases at 20 Hz. In the 30 Hz drive condition, a decrease was visible, in contrast to experimental data [4,5].

In conclusion, only prolonged GABAergic decay time constants (2), but not interventions (1) and (3) led to changes in entrainment comparable to experimental evidence in agreement with previous modeling approaches [5].

Our simulations suggest that prolonged time constants at GABAergic synapses might play a key role in abnormal evoked gamma rhythms in schizophrenia. However, since we only investigated one intervention at a time, further studies are needed to investigate the complex interactions of these circuit abnormalities. Furthermore, it remains unclear if the same mechanism also underlies increased spontaneous gamma activity in schizophrenia.
